# Transcriptional profiling of eosinophil subsets in interleukin‐5 transgenic mice

**DOI:** 10.1002/JLB.6MA1117-451R

**Published:** 2018-05-14

**Authors:** Kirsten A. Fairfax, Jessica E. Bolden, Aaron J. Robinson, Erin C. Lucas, Tracey M. Baldwin, Kerry A. Ramsay, Rebecca Cole, Douglas J. Hilton, Carolyn A. de Graaf

**Affiliations:** ^1^ Division of Molecular Medicine Walter and Eliza Hall Institute of Medical Research Parkville Australia; ^2^ Department of Medical Biology The University of Melbourne Parkville Australia

**Keywords:** gene expression, granulocyte, progenitor, Siglec‐F, trajectory

## Abstract

Eosinophils are important in fighting parasitic infections and are implicated in the pathogenesis of asthma and allergy. IL‐5 is a critical regulator of eosinophil development, controlling proliferation, differentiation, and maturation of the lineage. Mice that constitutively express IL‐5 have in excess of 10‐fold more eosinophils in the hematopoietic organs than their wild type (WT) counterparts. We have identified that much of this expansion is in a population of Siglec‐F high eosinophils, which are rare in WT mice. In this study, we assessed transcription in myeloid progenitors, eosinophil precursors, and Siglec‐F medium and Siglec‐F high eosinophils from IL‐5 transgenic mice and in doing so have created a useful resource for eosinophil biologists. We have then utilized these populations to construct an eosinophil trajectory based on gene expression and to identify gene sets that are associated with eosinophil lineage progression. Cell cycle genes were significantly associated with the trajectory, and we experimentally demonstrate an increasing trend toward quiescence along the trajectory. Additionally, we found gene expression changes associated with constitutive IL‐5 signaling in eosinophil progenitors, many of which were not observed in eosinophils.

## Abstract 2

Eosinophils in Interleukin‐5 transgenic mice can be subdivided by Siglec‐F expression, and are transcriptionally distinct.

**Abbreviations**
BM: bone marrow
CMP: common myeloid progenitor
DE: differentially expressed
EoH: Siglec‐F High Eosinophils
EoM: Siglec‐F Medium Eosinophils
EoP: eosinophil precursor
EPX: eosinophil peroxidase
GMP: granulocyte‐macrophage progenitor
GO: gene ontology
IL‐5Rα: IL‐5 receptor alpha
IL5T: IL‐5 transgenic
PB: peripheral blood
Spln: spleen
WT: wild‐type

## 1 INTRODUCTION

Eosinophils are granulocytes that play a role in the pathogenesis of asthma, atopic dermatitis, and allergy. Although they have been associated with defense against parasites such as helminths, it is their pathogenic inflammatory role that has significant implications for human health in the developed world.

Eosinophils are produced in the bone marrow (BM) and differentiate from myeloid progenitors in response to IL‐3, GM‐CSF, and more selectively, IL‐5.^1^, ^2^ Although eosinophils are normally a rare population, upon IL‐5 stimulation, which can occur in response to a parasitic infection and inflammatory disease, the BM can produce several orders of magnitude more eosinophils than are seen at steady state. Transgenic mice that constitutively over express IL‐5 (IL5T [IL‐5 transgenic]: IL‐5 is produced by T cells, under control of the CD2 promoter) have a permanent and extreme eosinophilia, where eosinophils make up over half of all leukocytes in the BM.^3^ IL‐5 acts at multiple levels throughout the eosinophil lineage, regulating eosinophil production, activation, migration, and survival.^3^, ^4^, ^5^, ^6^

Murine eosinophils can be identified using flow cytometry by their granularity and their expression of IL‐5 receptor alpha (IL‐5Rα) and the surface lectin Siglec‐F.^7^, ^8^ Siglec‐F possesses an ITIM characteristic of the Siglec family that can mediate inhibitory functions including induction of apoptosis.^9^ In mouse models of allergy in which Siglec‐F ligand has been deleted from bronchial epithelial cells and some immune cells, eosinophil populations are expanded due to reduced apoptosis.^10^ Following stimulation in a lung allergy model, Siglec‐F expression increases on eosinophils in BM, peripheral blood (PB), and spleen (spln), marking them for clearance.^11^ Siglec‐F has a functional analogue in humans—Siglec‐8—rather than a genuine paralogue, which convergent evolution has filling a similar role.^9^

Novel therapies targeting IL‐5 signaling are approved for the treatment of eosinophilic asthma,^12^, ^13^ and anti‐IL‐5 and anti‐Siglec‐8 therapies are currently in development for the treatment of diverse eosinophilic diseases.^14^, ^15^, ^16^

Here, we have studied the gene expression changes associated with IL‐5 up‐regulation. We noted the expansion of 2 eosinophil populations in IL5T mice that were distinguished on the basis of medium and high Siglec‐F expression. We transcriptionally profiled these populations in BM and blood and have made these transcriptional analyses available at haemosphere.org. Transcriptional profiling and in silico approaches were used to reconstruct the eosinophil lineage (from common myeloid progenitors (CMP) through to mature eosinophils), and show that the Siglec‐F^hi^ eosinophil population from the PB, which is rare in wild type (WT) mice, falls at the end of this linear trajectory. We identified gene sets that were associated with eosinophil lineage progression and increased Siglec‐F expression, which include eosinophil specific transcription factors and cell cycle regulators. Furthermore, eosinophils along this trajectory were shown to have demonstrably different cell cycle profiles and to trend toward quiescence. Finally, we explored the transcriptional consequence of constitutive IL‐5 signaling on the lineage, noting gene expression changes that were specific to eosinophil precursors (EoPs).

## 2 MATERIALS AND METHODS

### 2.1 Transgenic mice

All procedures involving animals were approved by The Walter and Eliza Hall Institute of Medical Research Animal Ethics Committee. IL5T mice were originally described in Ref. 3, where IL‐5 is constitutively expressed under the CD2 promoter. Mice that were used for transcriptomics were originally derived on a BALB/c background, while for the ELISA, cytocentrifuges, qRT‐PCR, and flow cytometry population quantifications they were backcrossed onto a C57BL/6 background for at least 9 generations. Fucci mice were originally described in Ref. 17 and were obtained from the Riken BioResource Centre.

### 2.2 Cell purification

All cells were purified from mice between 7 and 12 weeks of age. BM was collected from femurs and tibiae. PB was collected from the retro‐orbital sinus into Microtainer tubes containing EDTA (BD Biosciences). Single‐cell suspensions of Spln cells were prepared using a 100 μm cell strainer (BD Falcon). Red blood cells were lysed using an ammonium chloride buffer. Samples were stained with surface antibodies to Siglec‐F (BD E50‐2440), IL‐5Rα (BD T21), CCR3 (BioLegend Jo73E5), cKit (BD 2B8), CD34 (BD RAM34), Sca1 (D7), CD11b (in house M1/70), or a lineage cocktail of in‐house CD3 (KT31‐1), CD4 (GK1.5), CD8 (53‐6‐6), B220 (RA3‐6B2), Gr1 (RB68C5), and Ter119 (Ly‐76), then isolated on a fluorescence‐activated cell sorter. Stained cells were analyzed on a BD LSR Fortessa X‐20 flow cytometer (BD Biosciences) or sorted on a BD FACS Aria III (BD Biosciences). Re‐sort of sorted populations showed average purity of 91%. Cell populations for sorting for transcriptomics were defined using the following surface markers: eosinophils (IL‐5Rα Int, Siglec‐F high or medium), CMP (Lin–, cKit+, Sca1–, CD34+, and CD16/32lo), granulocyte‐macrophage progenitor (GMP; Lin–, cKit+, Sca1–, CD34+, CD16/32+, and IL‐5Rα‐),^18^ EoP (Lin–, cKit+, Sca1–, CD34+, CD16/32+, and IL‐5Rα+). For FACS analyses, EoPs were gated as per Supplementary Fig. 4 from Doyle et al.^19^ (Lin–, Sca1–, CD34+, cKit lo, and IL‐5Rα+), and eosinophil gating included the marker CD11b+. Prior to flow cytometry, cells were resuspended in PBS supplemented with 2% FCS, 2 mM EDTA, 1 μg/mL propidium iodide (Sigma) to enable identification and exclusion of dead cells.

Flow cytometric analyses were performed with FlowJo v10 software (FlowJo). Subsequent statistical tests and graphs were generated with Prism (GraphPad Software).

### 2.3 Data availability

All data can be viewed and are available for download on haemosphere.org. The dataset is also available in the Gene Expression Omnibus under the accession number GSE112010.

Full details of DNA content analyses, ELISA, microarray, cytocentrifuge, RNA isolation, RT qPCR, and bioinformatics analyses can be found in the supplementary methods.

## 3 RESULTS AND DISCUSSION

**Figure 1:**
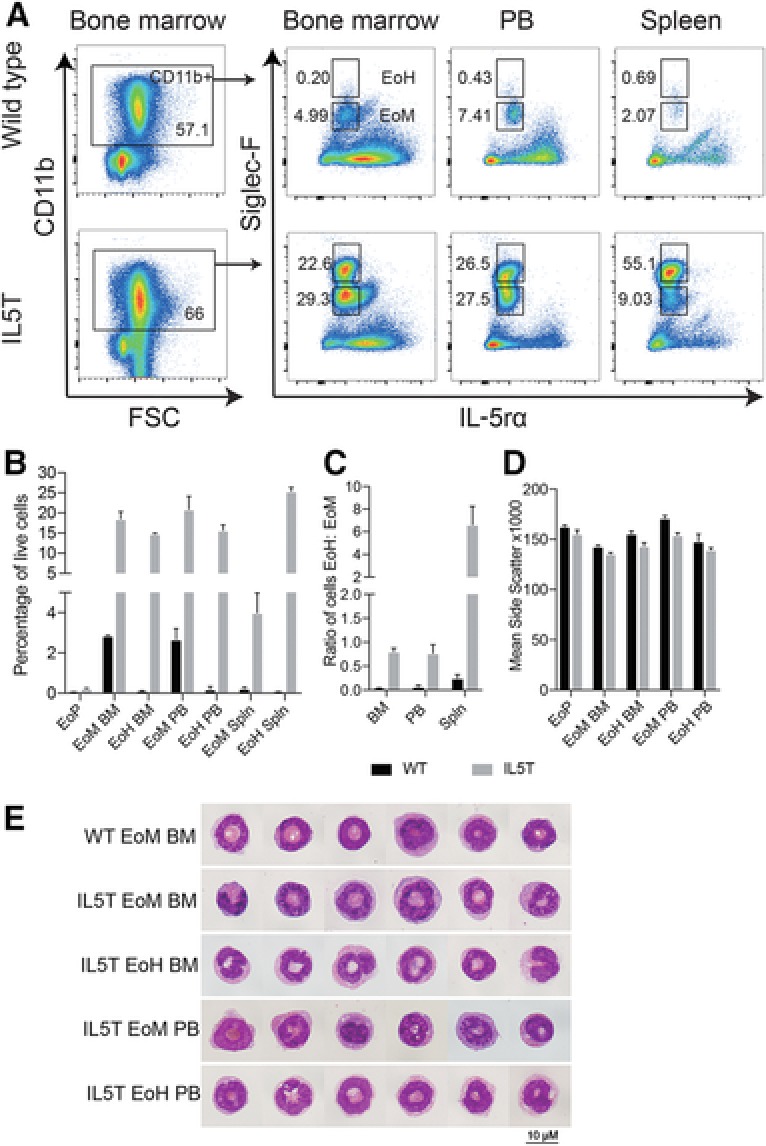
**Eosinophil populations in IL5T mice**. (A) Flow cytometry profiles of eosinophils collected from the BM, PB, and Spln of WT and IL5T mice. Populations have been gated for CD11b+ cells as shown. All mice are on a C57BL/6 background. (B) Proportion of eosinophil populations that make up the live cells in the BM, PB, and Spln. EoPs were gated for live cells then Lin–, Sca1–, CD34+, cKit lo, and IL‐5Rα+ 19as shown in Supplementay Fig. 1. *p < 0.05, *n* = 3, mean ± sd shown. (C) Ratio of EoH to EoM in different tissues, *n* = 3 for each genotype, Mean ± sd shown. (D) Side scatter of eosinophil populations, *n* = 3, mean ± sd shown. (E) May–Grünwald Giemsa stained cytocentrifuged preparations of WT and IL5T eosinophil populations viewed at 1000× magnification. Two representative cells were chosen from preparations from 3 separate mice for each population

Cells of the eosinophil lineage are rare in hematopoietic tissues of healthy WT mice but dramatically expanded in IL5T mice as has been reported by Dent et al.^3^ We confirmed this expansion in BM, Spln, and PB of IL5T mice, from EoPs to mature eosinophils (Fig. 1A and B). In isolating eosinophils for transcriptional analyses in both WT and IL5T mice, we observed eosinophils expressing medium levels of Siglec‐F (EoM) and a second population with high levels of cell surface Siglec‐F (EoH) (Fig. 1A). These 2 eosinophil populations were visible in the PB, Spln, and BM, and we sought to characterize them further.

The relative abundance of EoM and EoH varied dramatically depending on mouse genotype and tissue (Fig. 1B and C). In WT mice, eosinophils were rare, making up 2.7% of the nucleated cells in the PB, 0.22% in the Spln, and 2.9% in the BM, with EoM cells outnumbering EoH cells by a ratio of between 29:1 in the BM and 4:1 in the Spln. In IL5T mice, although both EoM and EoH were expanded in all 3 tissues, the effect was more dramatic for EoH cells, which was observed at similar levels to EoM in the BM and PB, and was 6‐fold more abundant than EoM cells in the Spln.

Flow cytometry analyses showed that side scatter, a measure of the granular complexity of cells, was high in EoPs and all mature eosinophil populations (Fig. 1D) irrespective of the level of Siglec‐F expression. To explore the morphology of these cells, we isolated EoM and EoH populations from the C57BL/6 IL5T BM and PB, and EoM from WT C57BL/6 BM by FACS and examined the cells following cytocentrifugation and May–Grünwald Giemsa staining (Fig. 1E). Morphologically, EoM and EoH resembled eosinophils, irrespective of the mouse genotype (C57BL/6 or IL5T) or the tissue from which they were isolated. All sorted populations had brightly staining eosinophilic cytoplasm with hypersegmented, ring‐shaped nuclei. We concluded that 2 populations of eosinophils were present in hematopoietic tissues of mice, which are identifiable on the basis of Siglec‐F expression and expanded in IL5T mice.

### 3.1 Cluster analysis of eosinophil and eosinophil progenitor expression profiles

Given that EoH were expanded upon constitutive IL‐5 stimulation (IL5T mice) and have been reported but not characterized in allergy models,^11^ it is possible they may play a role in disease. To explore the relationship between this population and other cells in the eosinophil lineage, we collected EoM and EoH from the BM and EoH from the PB of IL5T mice and analyzed gene expression using microarrays. We also collected EoPs, CMPs, and GMPs from WT and IL5T mice (Supplementary Fig. 1). These cells were all on a BALB/c background. We compared them to each other, and to transcriptional profiles of WT C57BL/6 eosinophils (bulk sorted without Siglec‐F substratification), EoPs, CMPs, and GMPs that have been previously published by our laboratory as part of a general survey of transcription in blood cells^20^ (Supplementary Table 1).

**Figure 2:**
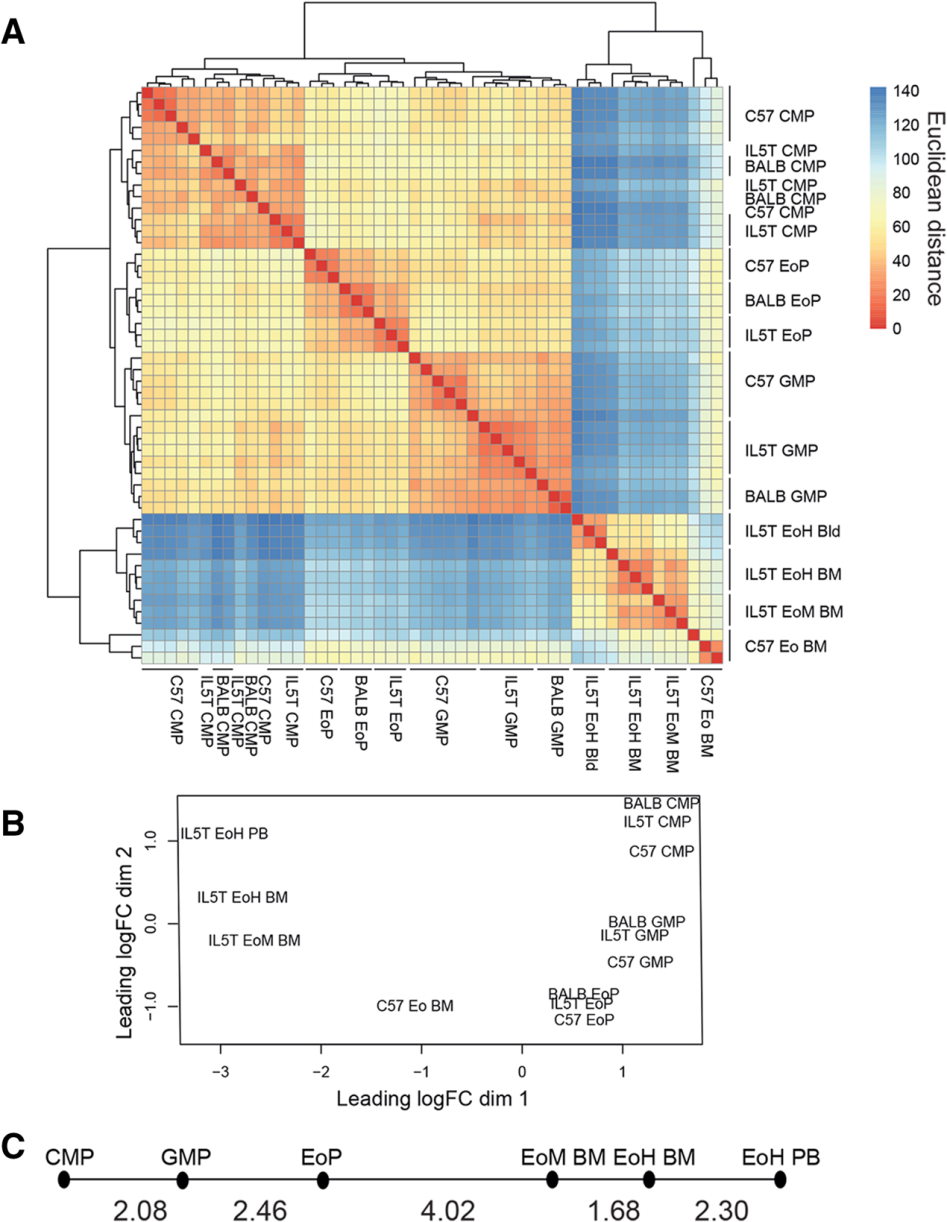
**Transcriptional profiling of eosinophils and progenitors**. (A) Hierarchical clustering of microarray samples using Euclidean distance. Each row and column represents an independent replicate. (B) Multidimensional scaling plot of average eosinophil and progenitor populations. The number of replicates for each group are as shown in (A). Strains and genotype of mice are C57 = WT C5LBL/6, BALB = WT BALB/c, and IL5T = IL5T BALB/c. (C) Minimum spanning tree of average gene expression in IL5T progenitors and eosinophil populations. Euclidean distance between the populations is shown

Hierarchical clustering of these samples showed that the progenitors all grouped by cell type, with little difference between “like” cell types from different genetic backgrounds (Fig. 2A). Eosinophils formed a separate cluster, within which the IL5T eosinophil samples clustered together by Siglec‐F expression and tissue of origin. C57BL/6 eosinophils formed a subcluster, rather than grouping with the IL5T EoM, to which they were closest by surface phenotype, a difference which may be driven by the effect of chronic IL‐5 stimulation on these cells.

Multidimensional clustering of the average expression for each cell type ordered the cells along a “reverse ‘U’‐shaped” trajectory, beginning with CMPs in the top right corner and ending with IL5T EoH cells from the PB at the top left (Fig. 2B). In these analyses, the C57BL/6 (C57) eosinophils BM were positioned between the EoPs and the IL5T eosinophils, with the IL5T EoM BM cells being most similar to the bulk sorted C57BL/6 BM eosinophils and the IL5T EoH PB terminating the series.

To independently confirm both the ordering of cell types and their arrangement in a linear trajectory, we created a minimum spanning tree from the IL5T samples. This is an unsupervised technique which links cell types with their nearest neighbor by Euclidean distance. Although the algorithm allows for branching between cell types, in our analyses we found the cell types formed a linear trajectory from CMP through to EoH PB (Fig. 2C). Based on these results, we propose an order for the IL5T eosinophil lineage: a linear trajectory from EoP to EoM BM to EoH BM to EoH PB.

Together, these data support the notion that transcriptionally, EoH are the terminal definable state in our eosinophil trajectory. We interpret this as EoHs representing activated or stimulated eosinophils, which are present in WT mice and driven by constitutive IL‐5 stimulation in IL5T mice.

### 3.2 Identification of eosinophil trajectory genes

**Figure 3:**
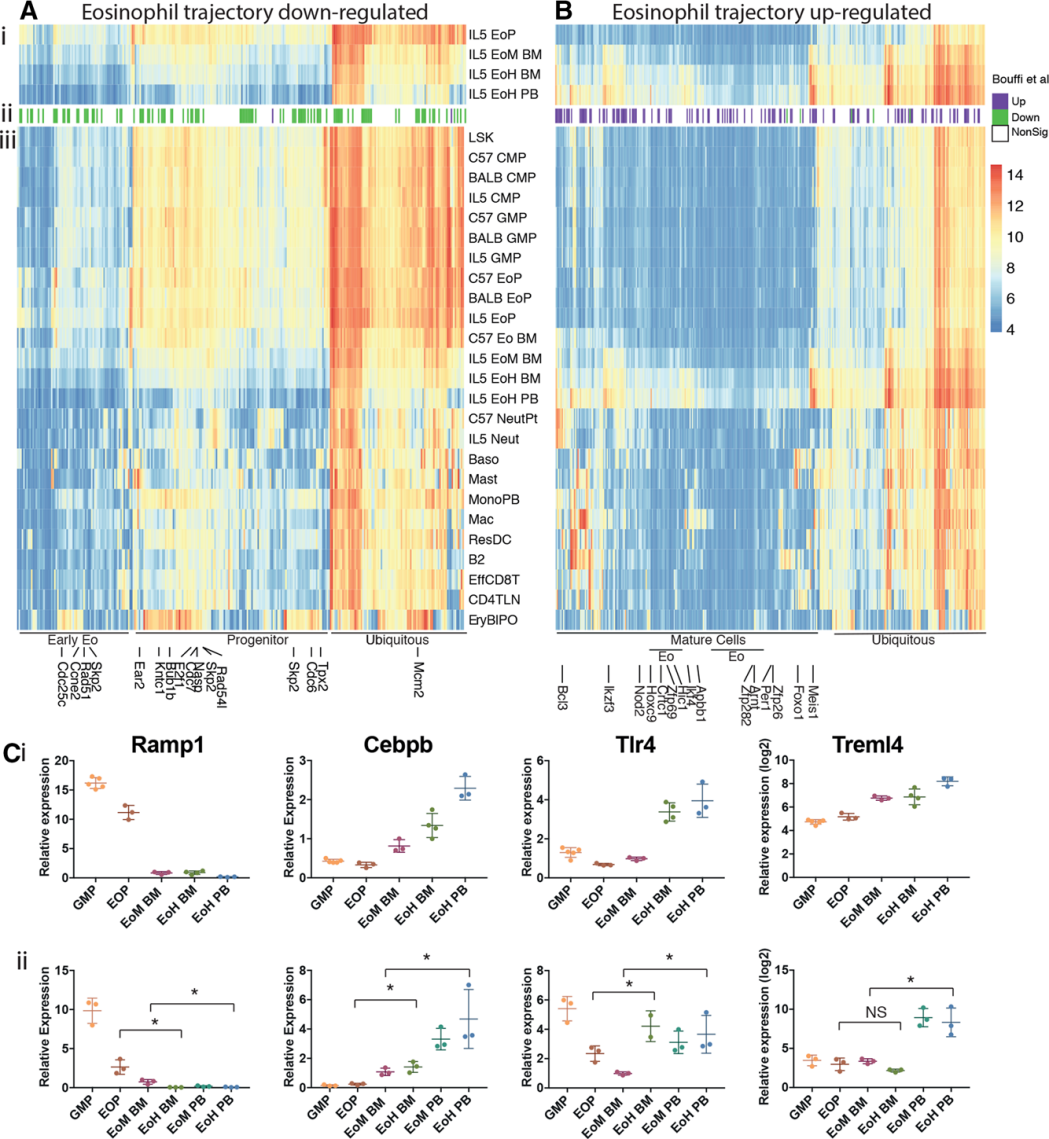
**Identification of genes up‐ or down‐regulated across the IL5T eosinophil trajectory**. Heatmap of genes that are (A) down‐regulated or (B) up‐regulated across the IL5T eosinophil trajectory. (i) The eosinophil populations used to select the genes of interest. Genes are significantly differentially expressed (DE) (log_2_FC > 0.5 and false discovery rate [FDR] < 0.05) in the same direction EoP versus EoH BM, and EoM BM versus EoH PB. (ii) DE genes (log_2_FC > 0.5 and an FDR < 0.05) in EoP versus eosinophils according to Bouffi et al.^21^ NonSig, not significantly different. (iii) Expression levels of up‐ and down‐regulated genes across hematopoiesis. Transcriptional profiles of other hematopoietic cell types are taken from Haemopedia.^20^ LSK = Lin‐Sca+Kit+ cell, NeutPt = peritoneal cavity neutrophil, Neut = bone marrow neutrophil, Baso = basophil, Mast = mast cell, MonoPB = monocyte from the peripheral blood, Mac = macrophage, ResDC = resident dendritic cell, B2 = B2 B cell, EffCD8T = effector CD8+ T cell, CD4TLN = CD4+ T cell from the lymph nodes, EryBLPO = ortho and poly erythroblasts. Where multiple classes exist for a cell type, the genotype and strain are highlighted. C57 = WT C57BL/6, BALB = WT BALB/c, and IL‐5 = IL5T BALB/c. All other cells are on a WT C57BL/6 background. Expression levels in (i) and (iii) are colored according to absolute expression of genes on a log_2_ scale. Gene sets with distinct expression patterns during hematopoiesis are highlighted. On (A) a granule protein gene, *Ear2*, and cell cycle genes are labeled on the plot, on (B) mature cell and eosinophil restricted transcription factors are highlighted. (C) qPCR validation of expression changes of selected genes from (A) and (B). (i) Expression levels in IL5T eosinophil populations from microarray data. (ii) qPCR expression levels. Scales are log or linear as indicated, depending on data range. Each dot represents an independent replicate (for microarray each replicate is a pool of mice, for qPCR each replicate is a single mouse). Mean ± sd. *p < 0.05

Having demonstrated the positioning of cells along a linear trajectory, we hypothesized that as cells progress along the series genes they would be gradually up‐ or down‐regulated. We have termed such genes “trajectory genes”. To identify trajectory genes, we focused on genes that were differentially expressed (DE) between (1) EoPs versus EoH BM cells and (2) EoM BM versus EoH PB from IL5T mice. These comparisons spanned the key “eosinophil specific” parts of our developmental progression—as opposed to including multi‐potential progenitors such as CMPs and GMPs. We did not compare the cells that were immediately adjacent to each other in the series because adjacent cells were likely to have smaller transcriptional differences and thus a higher background to signal ratio leading to a lower ability to detect key genes. We reasoned that this would identify key trajectory genes, without being unduly restrictive. We selected genes that were significantly up‐ or down‐regulated with a log_2_ fold change of at least 0.5. Using this approach, we identified 330 up‐regulated genes (353 probes; Fig. 3A(i)) and 271 down‐regulated genes (290 probes; Fig. 3B(i)) that were significantly altered across the eosinophil trajectory (Supplementary Tables 1 and 2). Expression profiles of 4 such trajectory genes (*Ramp1*, *Cebpb*, *Tlr4*, and *Trem14*) were validated using RT qPCR (Fig. 3C). Moreover, there was high concordance with our data and published WT BALB/c RNASeq data generated by the Fulkerson laboratory^21^ comparing EoPs to eosinophils (Fig. 3A(ii) and B(ii)). Of our 601 DE genes, 261 were significantly DE in the same direction with at least a log_2_ fold change of 0.5, and only 8 were significantly DE in the opposite direction (Fig. 3A(ii) and B(ii)). To investigate if trajectory gene expression changes were specific to eosinophils or occurred generally during hematopoietic differentiation, we examined their expression in a broad range of hematopoietic cells^20^ (Fig. 3A(iii) and B(iii)). Down‐regulated eosinophil trajectory genes generally had higher expression in progenitors than mature cells, implying general functions within progenitors and stem cells that are down‐regulated with differentiation into various lineages. Down‐regulated trajectory genes could be separated into 3 broad classes by expression pattern—those that were expressed in many cell types (ubiquitous), those that were expressed more highly in progenitors, and those that were expressed at higher levels in EoPs (denoted as early eosinophil, Fig. 3A(iii)).

We also examined the expression of up‐regulated eosinophil trajectory genes across wider hematopoiesis and found that the majority of them had their expression restricted to particular cell types. We grouped them into 3 categories: genes with ubiquitous expression, those that were more highly expressed in mature cell types, and those that were more restricted to eosinophils (Fig. 3B(iii)).

We further examined up‐regulated trajectory genes with restricted expression in mature cells and eosinophils as these were the most likely to have functional importance. In the mature cell set, we found 9 transcription factors. These included *Ikzf3* (Aiolos), *Nod2*, *Bcl3*, and *Foxo1*, which are reportedly induced in eosinophil maturation.^21^ *Ikzf3* binding sites have also been shown to be enriched in active genes regulated during eosinophil development.^21^

Six eosinophil specific transcription factors—*Arnt, Crtc1, Hic1, Hoxc9, Zfp689*, and *Zfp282*—were also found to be up‐regulated (Fig. 3B). *Crtc1* is known to be up‐regulated from WT EoPs to eosinophils^21^ and the other factors may have roles in IL‐5 response and the processes associated with eosinophil lineage progression.

**Figure 4:**
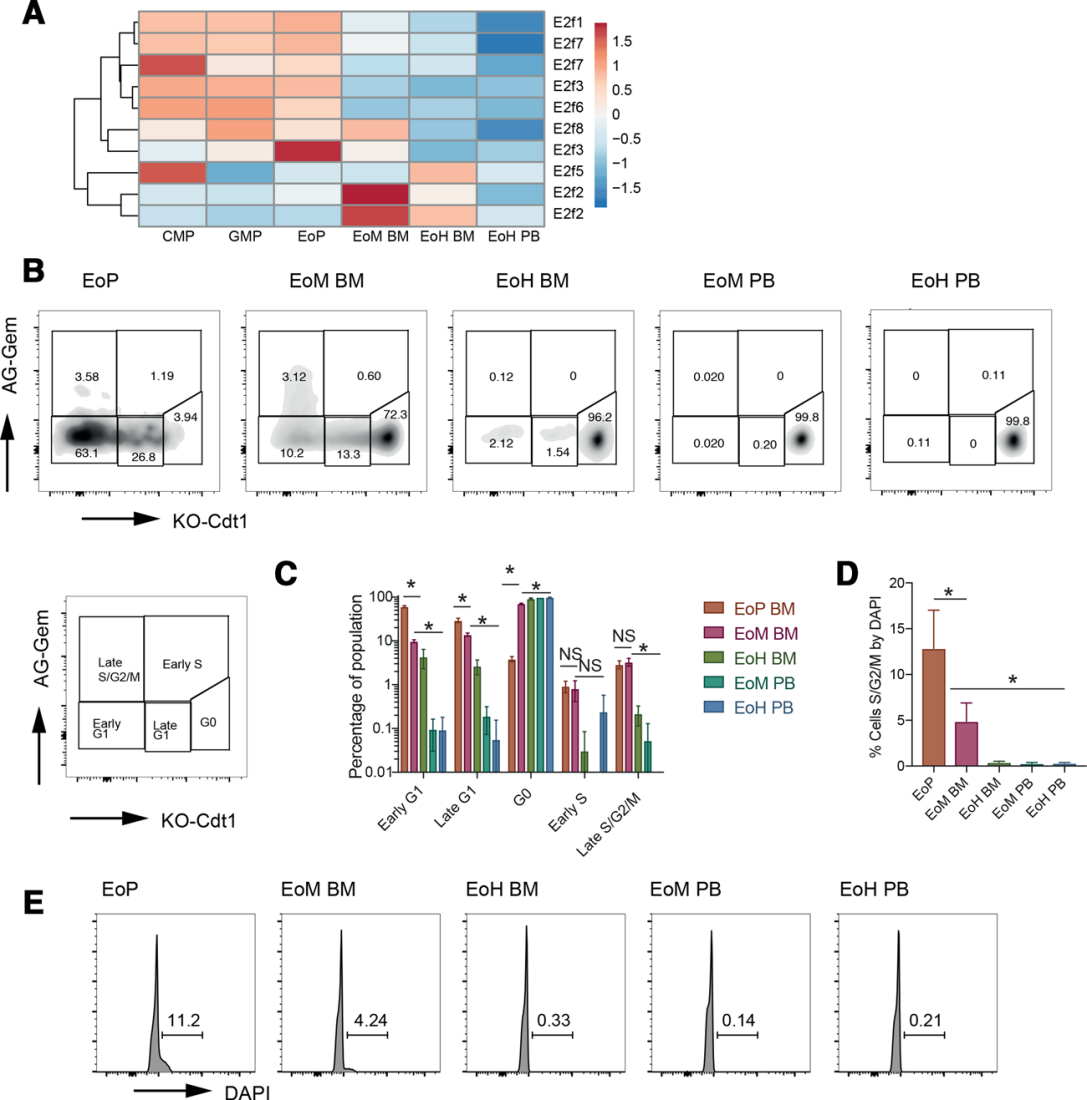
**Cell cycle status in eosinophil lineage populations**. (A) Relative expression of E2F family probes in BALB/c IL5T progenitors and eosinophils. Where gene names are shown twice, the gene was detected with multiple probes. Expression values are normalized to show relative expression of each probe and differences in gene expression are shown on a log_2_ scale. (B) Representative flow cytometry plots of eosinophil lineage populations from Fucci mice with proportions of cells in each gate. Schema of how staining corresponds with cell cycle stage as defined in Tomura et al.^25^ is shown. (C) Quantification of Fucci cell cycle data. Plots show mean ± sd (*n* = 3 indivdual mice). Significance is shown for EoP versus EoM BM and EoM BM versus EoH PB. *p < 0.05. (D) Quantification of S/G_2_/M phase (>2N DNA content) in eosinophil lineage populations by DAPI staining. Mean ± sd (*n* = 4 individual IL5T C57BL/6 mice). Significance is shown for EoP versus EoM BM and EoM BM versus EoH PB. *p < 0.05. (E) Representative histograms of DAPI staining levels by flow cytometry to indicate DNA content of each eosinophil lineage population. Peak represents 2N DNA content (G_1_/G_0_), with the marker covering >2N (S/G_2_/M phases)

To discover more about the function of eosinophil trajectory genes, we tested gene sets derived from gene ontology (GO) terms sourced from MSigDB^22^ with Fisher's exact test for overrepresentation. Multiple GO terms associated with the “cell cycle” (genes including *Cdk1, Cdca3*, and mini‐chromosome maintenance genes) were statistically overrepresented in down‐regulated trajectory genes, whereas “negative regulation of the cell cycle” (including the cyclin‐dependent kinase inhibitor encoding genes *Cdkn2a* and *Cdkn2b*) was overrepresented in the up‐regulated trajectory genes. Moreover, subsequent testing of trajectory gene sets for overrepresentation of transcription factor binding motifs revealed enrichment for the E2F family (Fig. 4A), known to play a key role in cell cycle regulation.^23^ Together, these data are consistent with cell cycle arrest and entry into quiescence across the eosinophil trajectory.

To test directly whether EoP, EoM, and EoH populations differed in terms of cell cycle in vivo, we assayed these populations by flow cytometry. In the WT context, we examined eosinophils from transgenic Fucci mice,^17^ in which fluorescent reporters had been linked to proteins Cdt1 and geminin that oscillate inversely during the cell cycle. Cdt1, linked to Kusabira Orange, accumulates during G_1_ and G_0_ and is promptly degraded at the onset of S‐phase. Geminin, fused to Azami‐Green, accumulates in S/G_2_/M phases, and is degraded at the completion of mitosis.^17^, ^24^ In Fucci mice, EoP, EoM, and EoH cells significantly differed in their cell cycle distribution (Fig. 4B and C). EoPs were mostly in early and late G_1_ (consistent with active, albeit slow cycling), whereas EoH in the BM and eosinophils in the PB were almost exclusively in G_0_ (consistent with cell cycle exit^25^). Interestingly, a clear population of cycling (S/ G_2_/M and early/late G_1_) EoM cells were present in the BM.

As IL5T mice were not available on the Fucci background, we examined the DNA content in IL5T eosinophil populations with DAPI to assess cell cycling (Fig. 4D and E). Again, we see that EoP and EoM in the BM have a significant population with >2N DNA content (denoting cells in S/G_2_/M phases), whereas EoH BM and PB eosinophils had almost exclusively 2N content and were likely in G_1_/G_0_ arrest. Together, these data demonstrate that cells become quiescent as they progress along the eosinophil trajectory, and that EoP, EoH, and EoM differ in terms of their cell cycle profiles.

### 3.3 Granule protein production in IL5T mice

**Figure 5:**
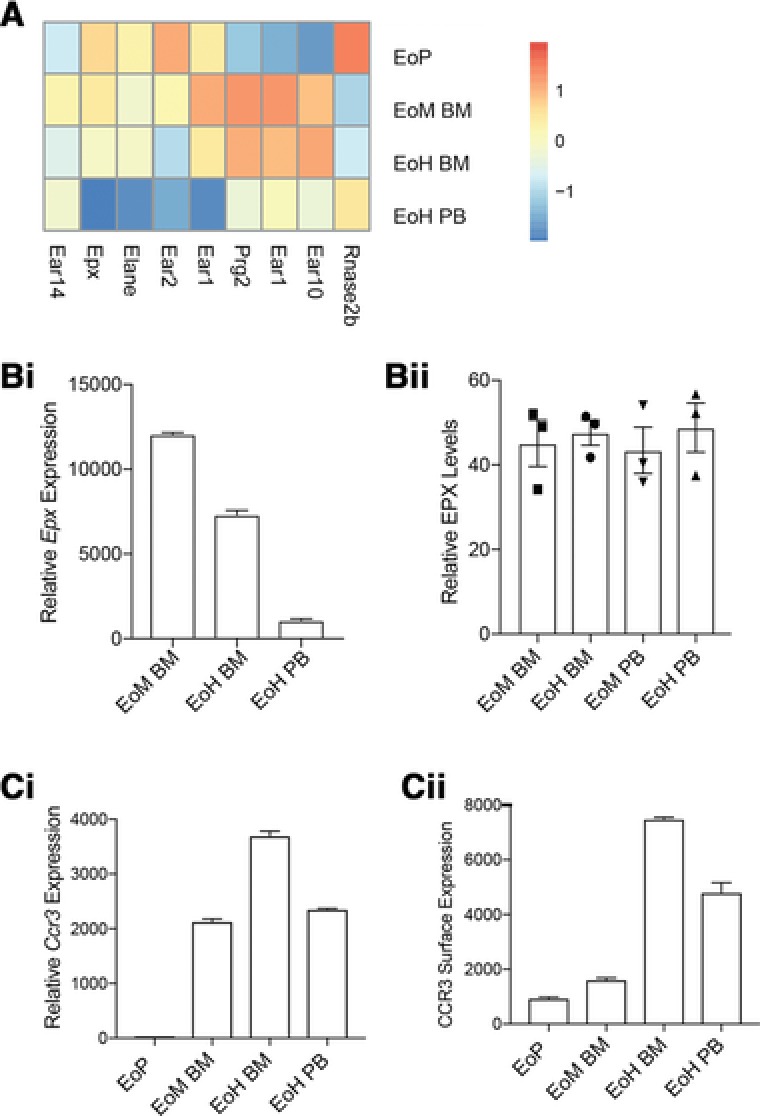
**Granule protein production in IL5T eosinophils**. (A) Heatmap of granule protein gene expression in IL5T eosinophils. Where gene names are shown twice, the gene was detected with multiple probes. *n* for each population as shown in Fig. 2A. Expression values are normalized to show relative expression of each gene and differences in gene expression are shown on a log_2_ scale. (B) Comparison of (i) *Epx* expression in IL5T eosinophil populations from microarray data. (Values are shown on a linear scale) to (ii) ELISA of EPX production by IL5T eosinophil populations showing relative EPX concentration in each population. Error bars show mean ± sem. (C) Comparison of (i) *Ccr3* transcript levels measured by microarray to (ii) CCR3 surface protein levels measured by flow cytometry. Mean + sem

The production and release of granule components, including the cytotoxic proteins eosinophil peroxidase (EPX), major basic protein, and eosinophil associated RNAses, are central to eosinophil function. Analysis of transcript expression for genes associated with granules in IL5T mice (Fig. 5A) peaks when cells are in the BM, consistent with that reported in WT mice.^21^ For some granule genes (*Rnase2b*, *Ear2*, and *Epx*), transcription peaks in EoPs and markedly drops as cells progress through the trajectory, whereas other granule components (*prg2* and *Ear1*) reach peak expression in EoM BM.

To compare the pattern of transcript production to protein storage, we performed an EPX ELISA on IL5T eosinophil populations (Fig. 5B). EPX protein levels were not significantly different between these populations. This supports the notion that EPX production occurs early in eosinophil development in the BM and is then stored until it is required in the periphery. This is in contrast to the expression of the CCR3, the eotaxin receptor, which has the levels of protein surface expression more correlated with its transcript levels (Fig. 5C).

### 3.4 Effects of constitutive IL‐5 on EoPs

**Figure 6:**
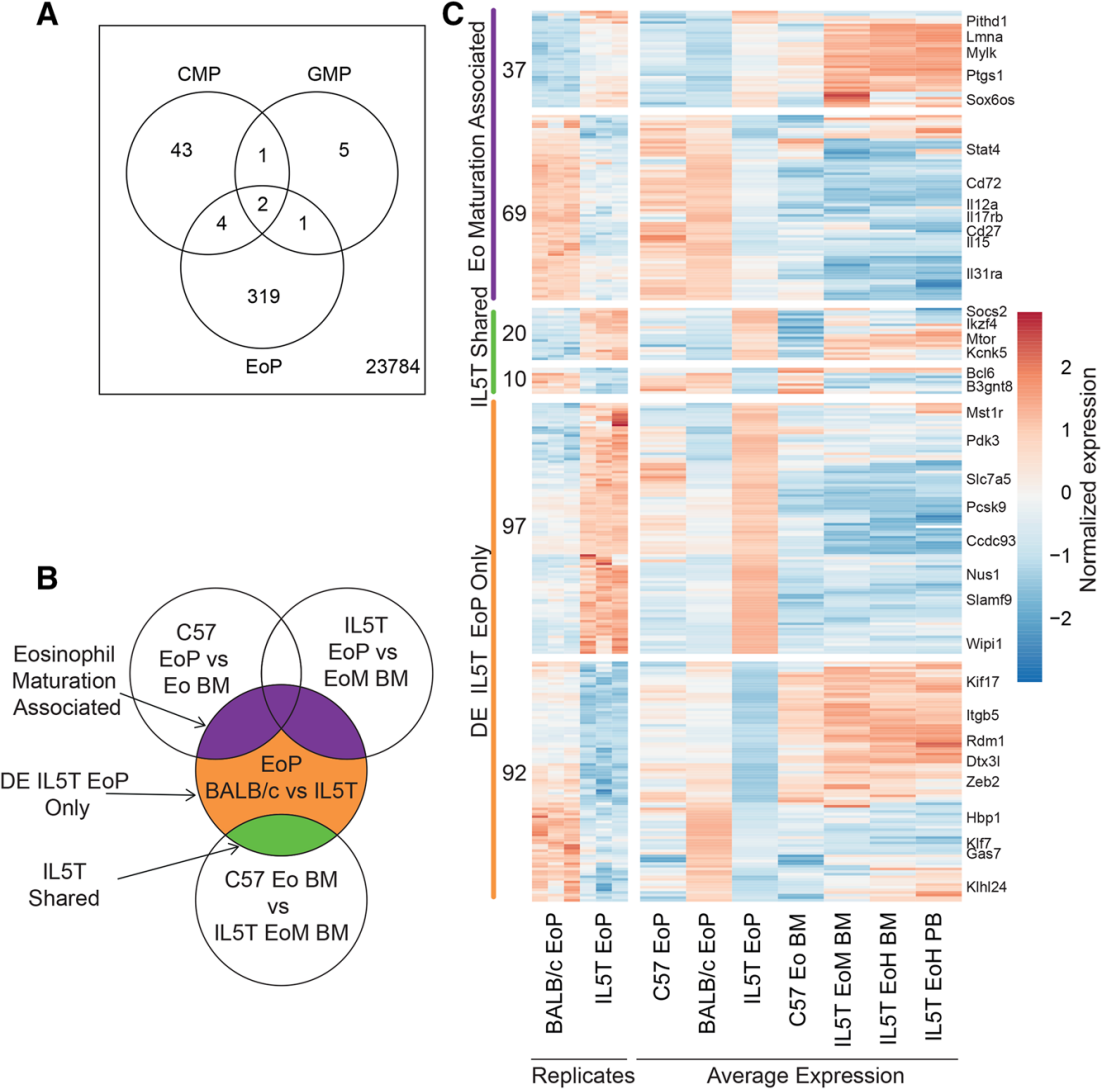
**Transcriptional differences between wild type (WT) and IL5T progenitors**. (A) Venn diagram showing overlap of differentially expressed (DE) genes (FDR < 0.05) between WT BALB/c and IL5T BALB/c progenitor populations. (B) Schema showing division of IL‐5 responsive genes in EoPs (BALB/c EoP v IL5T BALB/c EoP) into 3 groups as shown on the Venn diagram. (C) DE genes between WT BALB/c and IL5T BALB/c EoPs. Expression in individual replicates of these cell types is shown to the left. Average expression in relevant cell types for these comparisons is shown to the right. Genes are grouped according to the scheme in (B). Numbers of probes in each group are listed on the left and select gene names are shown on the right. Full details of genes are given in Supplementary Tables 1 and 3. Heatmap is colored according to mean normalized expression. Genotypes labeled BALB/c = WT BALB/c, C57 = WT C57BL/6, and IL5T = IL5T BALB/c. FDR < 0.05 for all comparisons

EoPs express IL‐5Rα and are responsive to IL‐5 stimulation. Gene expression changes have been examined in BM cells cultured in vitro with IL‐5,^26^ but little is known about the transcriptional differences caused in eosinophil progenitors by chronic IL‐5 stimulation in vivo. We therefore compared the transcription profiles of EoPs, CMPs, and GMPs isolated from IL5T to their counterparts from WT mice. In EoPs, the influence of IL‐5 stimulation was prominent with 325 DE probes (Fig. 6A), as opposed to the few significantly DE genes in the CMPs and GMPs (51 and 11 probes, respectively, at a false discovery rate of <0.05), suggesting IL‐5 stimulation has a major influence on cells committed to the eosinophil lineage.

We examined the expression of EoP IL‐5‐responsive genes in the eosinophil‐committed section of the eosinophil trajectory (from EoP to EoH PB; Fig. 6B and C). There were 3 major groups of genes (Fig. 6C): (1) those that were DE from EoP to eosinophils, which we considered associated with maturation, (2) those that were generally IL‐5 responsive, that is, not DE during maturation but also DE between WT and IL5T eosinophils—termed IL‐5 shared, and (3) those which did not fall into either previous category, so were only responsive to IL‐5 in EoPs (Supplementary Tables 1 and 3).

More than half the differential expression (58%) is due to specific effects of IL‐5 on EoPs, with few genes DE due to a general IL‐5 effect on both EoPs and eosinophils. A total of 33% of the EoP IL‐5 responsive genes were associated with eosinophil maturation including down‐regulation of cytokine signaling genes *Il12a*, *Il17rb*, *Il15*, *Il31ra, Cd72*, and *Stat4*.

Notably, genes involved in granule production were not significantly affected by IL‐5 stimulation in EoPs, despite being an early sign of EoP differentiation from less committed progenitors.

In summary, our data show that there are 2 different populations of eosinophils in IL5T and WT mice by Siglec‐F expression, which are transcriptionally distinct and differ in cell cycle profile. These populations follow a linear trajectory as assessed by transcriptional changes from EoP to EoM to EoH in the BM, and then to EoH in the periphery, and even among the eosinophils there is a gradient of cell cycle status, with cells trending toward quiescence. Our analyses provide a key platform for discovery of potential eosinophil lineage‐specific genes (such as the transcription factors *Arnt, Hic1*, *Hoxc9*, *Zfp689*, and *Zfp282)* that have been revealed by assessing the transcriptional changes that occur across the eosinophil trajectory.

Our dataset is presented as a resource to the eosinophil community and it enables comparison of several eosinophil and progenitor subsets on different genetic backgrounds. We have provided the outcomes of relevant comparisons in Supplementary Tables 1–3. Raw transcriptional data are available to download from GEO (GSE112010) or it can be directly visualized and compared to a comprehensive collection of hematopoietic cell types from diverse lineages at haemopedia.org.

## DISCLOSURE

This work was partially supported by funding from CSL to D.H

## Supporting information

Supplementary Methods

tableS1

tableS2

tableS3
