# Supplementary material for: Transcriptional profiling of eosinophil subsets in interleukin‐5 transgenic mice
Source: J Leukoc Biol. 2018 May 14;104(1):195–204. doi: 10.1002/JLB.6MA1117-451R (PMC6749942; doi:10.1002/JLB.6MA1117-451R)
Supplement: Supplementary file 1 — Supplementary Methods [file JLB-104-195-s001.pdf]

Supplementary Methods

DNA content analyses
Bone marrow and blood cells were red cell lysed and stained for surface markers as described in the "Cell purification" section of the methods. Stained cells were fixed and permeabilised in 250µL cytofix/cytoperm solution (BD) for 30 minutes on ice, washed twice with 1x perm/wash solution (BD). 200µL DAPI working solution (10µg/mL DAPI in 1x perm/wash) was added per $2x10^6$ stained cells, and incubated at room temperature for 30 minutes. Cells were washed with >10 volumes of 1x perm/wash, resuspended at $2x10^7$ cells per mL in perm/wash, filtered and analysed on a BD LSRII flow cytometer (BD Bioscience).

Cytocentrifuge preparations
Cytocentrifuge samples (between 20000 and 100000 cells) were spun onto Superfrost slides (Thermo Scientific – Menzel Glaser) at 500rpm, with low acceleration then air-dried. Slides were fixed for 10 minutes in methanol, then stained for 5 minutes in May-Grunwald's stain (Merck) followed by 20 minutes in 5% Geimsa stain (Merck) made up with pH 6.8-buffered water (Merck). Finally, slides were washed for 1 minute with gentle agitation in pH-6.8 buffered water, followed by 2x 30 second washes in deionised water. Slides were then air-dried and coverslipped (Menzel-Glaser). Images were acquired on a Nikon 90i microscope using a 100x objective and Nikon Digital Camera DXM1200C. Image analysis was performed using Adobe Photoshop.

EPX ELISA
Sorted cells were lysed in 0.22% hecadecyltrimethylammonium bromide (Sigma H5882)/0.3M sucrose (Fluka 84100) buffer at a concentration of 600,000 eosinophils per 0.5mL buffer, then ELISA performed using detection antibody, anti-EPX-bi (MM25-82.2.1-bi) according to method outlined in [1].

Transcript analysis by reverse transcription (RT) and qPCR
Total RNA was prepared using Qiagen RNeasy Mini or Micro kit according to manufacturer's instruction. cDNA was generated by reverse transcription using the SuperScript III First-Strand Synthesis System (Invitrogen) according to the manufacturer's instructions. qPCR was performed using FastStart Essential DNA Probes Master (Roche), PCR primers, and hydrolysis probes from the Universal ProbeLibrary (Roche) in a LightCycler 480 (Roche). The transcripts of the housekeeping gene Vamp3 were used for normalization of the samples.

RNA Isolation, amplification, and hybridization for microarrays
Total RNA was isolated from purified cell populations using RNeasy Micro/Mini kits (Qiagen). Each purified RNA sample was assessed for quality and integrity using the 2100 Bioanalyzer (Agilent Technologies).

RNA was amplified according to the manufacturer's instructions with the Illumina Total Prep RNA Amplification Kit (Ambion). The quality of the labeled product was again ascertained using the Agilent Bioanalyzer 2100. Labeled cRNA

was then hybridized to Illumina MouseWG-6 V 1.1 or 2.0 Expression BeadChips according to the manufacturer's instructions at the Australian Genome Research Facility.

Microarray analysis
We restricted our microarray analyses to a curated selection of probes that were supported by an Ensembl transcript [2] and that were found on both V1.1 and V2.0 beadchips. This resulted in 24159 probes covering 16973 genes. The dataset was normalized with the normexp background correction followed by quantile normalization [3]. A batch correction based on the beadchip version was applied to the linear model for plotting and differential expression analyses. Pairwise comparisons were made using linear modeling and empirical Bayes moderated t statistics [4]. The false discovery rate (FDR) was controlled by using the Benjamini-Hochberg algorithm [5]. Probes with FDR < 0.05 were considered to be differentially expressed. The multidimensional scaling plot of distances between cell types was made using the plotMDS function in the *limma* package in R [6]. Heatmaps were generated using the *pheatmap* package in R with clustering by the "complete" method [7].

Clustering
Samples were clustered using only genes with variance > 2. Distance between samples was calculated with Euclidean distance with these genes. Multidimensional Scaling plots (MDS plots) were calculated using the plotMDS function in *limma* [6]. After averaging the samples, the algorithm selects the top 500 genes by standard deviation between each pairwise comparison of cell types. These were used to calculate the Euclidean distance, which is used to form the plot.

RNASeq Analysis
Aligned counts from accession number GSE69707 [8] were obtained from the Gene Expression Omnibus [9]. Where there were multiple transcripts for a single gene, the transcript with the highest average expression across all cell types was selected. Differential expression analysis utilised the edgeR [10] and limma [6] software packages in R. Genes were selected as differentially expressed if they had an adjusted p value < 0.05 after Benjamini-Hochberg correction and log2 fold change < 0.5.

Gene set testing
Gene sets were downloaded from the Molecular Signatures Database (www.broadinstitute.org/gsea/msigdb) [11]. Human gene identifiers were mapped to mouse orthologues as described in [12]. Enriched gene sets were identified with Fisher's exact test, and p values were adjusted with Benjamini-Hochberg correction [5]. Gene sets listed had an adjusted p values < 0.05.

Minimum Spanning Tree
When forming the minimum spanning tree [13], the data was averaged for each cell type and top 500 genes with the highest standard deviation between each pair of samples was selected with the "pairwise" setting of plotMDS from the R package *limma* [6]. The Euclidean distance between each cell type was calculated

with these probes and this value was used to determine the minimum spanning tree with the *vegan* package in R [14].
In brief, a minimum spanning tree (MST) is calculated by assuming that cell types that have gene expression profiles that are very similar to each other are closely related, and we have used this information to infer the relationships between our cell types. An MST is a type of graph that is able to connect the cell types together in such a way that the connections between them are minimum. We have calculated the distance between the cell types based on Euclidean distance, and connected all the cells without loops so that the total distances used are minimum.

1. Ochkur, S. I., Kim, J. D., Protheroe, C. A., Colbert, D., Condjella, R. M., Bersoux, S., Helmers, R. A., Moqbel, R., Lacy, P., Kelly, E. A., Jarjour, N. N., Kern, R., Peters, A., Schleimer, R. P., Furuta, G. T., Nair, P., Lee, J. J. & Lee, N. A. (2012). A sensitive high throughput ELISA for human eosinophil peroxidase: A specific assay to quantify eosinophil degranulation from patient-derived sources. *J. Immunol. Methods* **384,** 10–20.

2. Barbosa-Morais, N. L., Dunning, M. J., Samarajiwa, S. A., Darot, J. F. J., Ritchie, M. E., Lynch, A. G. & Tavaré, S. (2009). A re-annotation pipeline for Illumina BeadArrays: Improving the interpretation of gene expression data. *Nucleic Acids Res.* **38,** e17.

3. Shi, W., Oshlack, A. & Smyth, G. K. (2010). Optimizing the noise versus bias trade-off for Illumina whole genome expression BeadChips. *Nucleic Acids Res.* **38,** e204.

4. Smyth, G. K. (2004). Linear models and empirical bayes methods for assessing differential expression in microarray experiments. *Stat. Appl. Genet. Mol. Biol.* **3,** Article3.

5. Benjamini, Y. & Hochberg, Y. (1995). Benjamini Y, Hochberg Y. Controlling the false discovery rate: a practical and powerful approach to multiple testing. *J. R. Stat. Soc. B* **57,** 289–300.

6. Ritchie, M. E., Phipson, B., Wu, D., Hu, Y., Law, C. W., Shi, W. & Smyth, G. K. (2015). limma powers differential expression analyses for RNA-sequencing and microarray studies. *Nucleic Acids Res.* **43,** e47.

7. Kolde, R. (2015). pheatmap: Pretty Heatmaps R Package version 1.0.8.

8. Bouffi, C., Kartashov, A. V., Schollaert, K. L., Chen, X., Bacon, W. C., Weirauch, M. T., Barski, A. & Fulkerson, P. C. (2015). Transcription Factor Repertoire of Homeostatic Eosinophilopoiesis. *J. Immunol.* **195,** 2683–

2695.

9. Edgar, R., Domrachev, M. & Lash, A. E. (2002). Gene Expression Omnibus: NCBI gene expression and hybridization array data repository. *Nucleic Acids Res.* **30,** 207–210.

10. Robinson, M. D., McCarthy, D. J. & Smyth, G. K. (2009). edgeR: A Bioconductor package for differential expression analysis of digital gene expression data. *Bioinformatics* **26,** 139–140.

11. Liberzon, A., Subramanian, A., Pinchback, R., Thorvaldsdóttir, H., Tamayo, P. & Mesirov, J. P. (2011). Molecular signatures database (MSigDB) 3.0. *Bioinformatics* **27,** 1739–1740.

12. de Graaf, C. A., Choi, J., Baldwin, T. M., Bolden, J. E., Fairfax, K. A., Robinson, A. J., Biben, C., Morgan, C., Ramsay, K., Ng, A. P., Kauppi, M., Kruse, E. A., Sargeant, T. J., Seidenman, N., D'Amico, A., D'Ombrain, M. C., Lucas, E. C., Koernig, S., Baz Morelli, A., *et al.* (2016). Haemopedia: An Expression Atlas of Murine Hematopoietic Cells. *Stem Cell Reports* **7,** 571–582.

13. Prim, R. C. (1957). Shortest Connection Networks And Some Generalizations. *Bell Syst. Tech. J.* **36,** 1389–1401.

14. Oksanen, J., Blanchet, F. G., Kindt, R., Legendre, P., Minchin, P. R., O'Hara, R. B., Simpson, G. L., Solymos, P., Stevens, M. H. H. & Wagner, H. (2015). vegan: Community Ecology Package. R package version 2.3-1. *http://CRAN.R-project.org/package=vegan*

Supplementary Figure 1

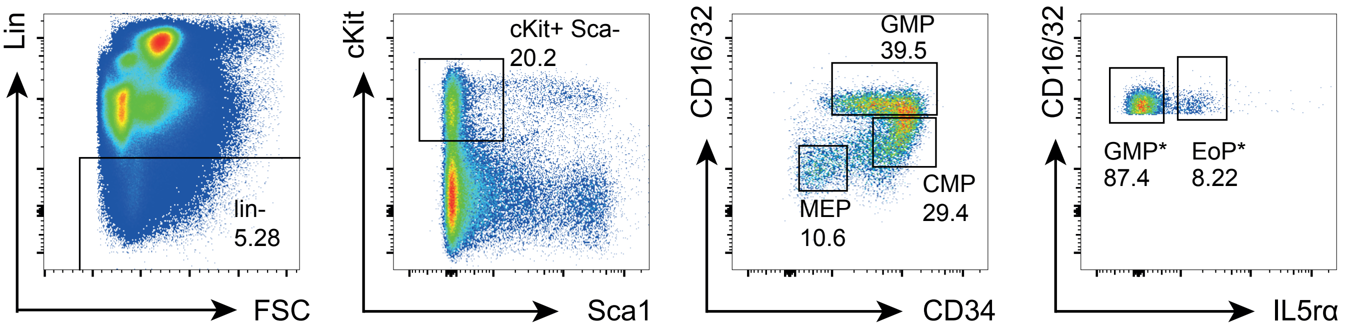

Supplementary Figure 1: Gating scheme of progenitors for transcriptional analysis
Scheme showing the gating strategy for CMPs, GMPs and EoPs for transcriptional analysis. Cells have been pregated to exclude doublets, debris and dead cells. Initial gates are set according to Akashi et al (2000), with the GMPs further divided into GMP* and EoP* using IL5Rα. This gating strategy was used to ensure that GMPs and EoPs did not overlap.
